# The Pathology of Joint and Bone Tuberculosis

**Published:** 1888-12

**Authors:** Weller Van Hook

**Affiliations:** 884 West Madison Street


### The Medical Journal and Examiner.

VOLUME LVII. DECEMBER, 1888. NUMBER 6.

### ORIGINAL COMMUNICATIONS.

### THE PATHOLOGY OF JOINT AND BONE TUBERCULOSIS.

BY WELLER VAN HOOK, A. B., M. D.

The pathology of tuberculosis has been, within recent years, much elucidated by studies founded upon the knowledge that the disease was, under all circumstances, the result of the hostile action of the specific bacillus of tuberculosis. When this microbe's well-known reaction to some ot the aniline dyes was promulgated, pathologists endeavored to ascertain the methods by which infection was effected, the position of the microbe in the invaded tissues, its action on the cells and fluids, and finally its own ultimate fate. The field of minute anatomical investigation, thus enlarged, was still further extended by the suggestive labors of Flemming, Retzius, Fol, Rabl, and others in the study of the methods of cell-multiplication, and especially the "indirect method" through the process of karyokinesis, the most common mode of reproduction, by the primary division of the nucleus after a series of changes in the most active part of the protoplasm, the "chromatin," or tangible part. The study of tuberculosis in its gross relations is so much simplified by a knowledge of these processes that it will here be preceded by a short review of the subject of tubercle-genesis.

Baumgarten was one of the most diligent investigators in the enlarged field of study opened up by the investigations of Koch and Flemming and their followers. His researches were made chiefly upon inoculation tuberculosis, often using the anterior chamber of the rabbit's eye. On injecting cultures of bacilli into the anterior chamber, it was found that after five days bacilli had found their way into the iris. After their invasion of the iris, cell-proliferation was demonstrable. This cell-proliferation is the first step in the formation of the tubercle after the bacteria have reached the nidus required. It involves the fixed connective-tissue corpuscles, which undergo karyokinetic changes, taking on the reproductive activity of embryonal connective-tissue, and form, by thus rapidly multiplying, heaps of so-called epithelioid cells.

The first step in the process consists in the increase in volume of the active cell. At the same time the entire cell becomes turgid and more rounded. The nucleus, whose chromatin in the resting state presents the appearance of a simple framework, enlarges and begins to exhibit active changes in its chromatin preparatory to division. The nucleus having divided, two daughter-cells are formed by the division of the cell-body. After reproduction has thus begun, it continues in the same tubercle, by repeated division of the peripheral epithelioid cells of the mass. The bacilli are not numerous within the dividing cells, but accumulate in the cells that are at rest. They are also found in abundance in the inter-cellular lymph-spaces.

Multinuclear giant-cells may or may not be formed at the centre of the nodule, probably from the division of the nuclei of the fixed cells. Vascular changes are brought about early. Vessel walls are attacked and their endothelium proliferates. A true inflammation being present, we find, as would be expected, an abundant emigration of leucocytes, which form a more or less compact zone of so-called lymphoid or round cells about the periphery of the tubercle. This inflammatory product may be so abundant as to obscure all other elements, thus producing what is knowu as a "small-celled" tubercle.

Despite the fact that the blood vessels are so early affected, no new vessels are formed and the tubercle remains avascular. Sooner or later the regressive changes known as coagulation necrosis, due to lack of nutrition, begin in the round cells, whose nuclei shrink, break down, and become fluid. Then the epithelioid cells die, become homogeneous, lose their nuclei, and become pale, glistening masses. In the giant-cells partial necrosis sometimes takes place, which, according to Weigert, is recognizable by diminution of staining capacity. The nuclei are then to be seen in one part of the cell, as at a single pole, at one side of the cell, about the whole circumference, or even in its centre only. The bacilli are often found at the junction of the living with the dead protoplasm, and may lie even in the non-nuclear part. Finally, such changes taking place in the individual cells, the whole tubercle can become a single homogeneous mass, or a collection of smaller masses, each representing a single cell, or even a granular mass which contains more or less fat. Thus, the tubercle loses its gray translucent quality and becomes an opaque yellowish body, which, by an exaggeration of the same process, may result, in combination with a quantity of serous exudate, in an emulsion-like fluid which, in large collections mixed with leucocytes and débris of connective tissue, is known as the "pus" of cold abscesses.

The contest, therefore, between the living active tissue-elements on the one hand—an army representing physiological resisting power—and, on the other hand, the hosts of microbic invaders standing for the advancing disease, can not be considered unreal. Indeed, it must be taken into consideration if we would understand the probable cause of the varying course of tubercular joint and bone disease. Schüller has proved experimentally that the microbes, having effected an entrance into the blood by way of the lungs, may be carried to parts remote. When such a mass of inimical foreigners has reached a situation favorable to the physiological functions of the bacillus, a multiplication of bacilli takes place with great rapidity. It is at this stage that the factor of local tissue-resistance and that of the invading forces are to be opposed to one another. On the one hand are the microbe's power of marvelously rapid multiplication; his ability to transform soluble albuminoids into the chemical constituents of his own body; his probable power of throwing off an alkaloidal waste-product that has a toxic action upon the tissues of the patient; his ability to resist alterations of temperature far greater than those to which his own activity excites his host; and finally, the almost incredible faculty of entering as a last resort that still lower condition of life known as the spore form, in which he can patiently await, oblivious to all but the most severe forms of external opposition, a better opportunity for self-multiplication. On the other hand, we have the cellular elements of the animal body, especially the leucocytes and the fixed connective-tissue corpuscles, the latter capable of taking upon themselves a state of active embryonic proliferation, aided by a more or less active circulation of nutrient fluid.

Such, we may say, are the abstract factors in the struggle, the advantages of the opposed individuals.

But there are other subtler and, as yet, ill-understood moments affecting the activity of each element that can not be ignored. The significant labors of Pasteur in the diminution of the virulence of several microbes by special methods of culture indicate the probable existence of varying degrees of invasive and resisting activity on the part of micro-organisms in general. And these observations on the limitations of microbic virulence find a striking analogy on the part of the animal tissues in the long observed clinical fact that each individual of our species presents a resisting power to tuberculosis peculiar to himself. When our knowledge of the tubercle bacillus is more extensive and exact, these considerations will, doubtless, find an abundant and important application in the study of the invasive powers of tuberculosis. Koenig, probably the most experienced of clinical surgeous in respect to this disease, has divided cases of tuberculosis in bones and joints into two classes—first, that "dry granulating form," in which caseation is retarded or absent, and peripheral extension is limited by fibrillation of the newly formed connective tissue, in a word, the cases tending to recovery; and second, the "moist form" of the disease, in which fluids are in excess and the disease advances rapidly by peripheral infection. It is to be regretted that pathologists have not furnished us as yet with any conclusive evidence as to which of the two forces, invading or resisting, is responsible in individual cases for this formation of "cold abscesses" in some instances, and "dry granulation-mass tuberculosis," in others. For the present, we can only refer to the general considerations mentioned, and say vaguely with Koenig that cases have tendencies to this or that form.

PRIMARY TUBERCULAR SYNOVITIS.—That minute masses of foreign material could be deposited in joint cavities after traumatism was proved by Schüller, as follows: A rabbit or goat having been selected as the animal for experimentation, a knee-joint was bruised with a few slight blows of a mallet, and at the same time an injection was made into the trachea or a large venous trunk of a quantity of finely divided, aseptic coloring matter. The pathological changes produced by a simple contusion depend on the amount of force and the manner of its application. The most constant and significant change is the occurrence of hæmorrhages into the articular and peri-articular structures. When injections of coloring matter were made simultaneously this matter was found on post-mortem examination in the effused blood and was directly proportionate in quantity to the degree of hæmorrhage. Such injuries to blood-vessels are followed by simple inflammatory reaction of a reparative character. Vascular dilatation, local œdema, and emigration of leucocytes are constant factors. If no foreign material is present except the finely-divided aseptic coloring matter recovery is rapid. Proliferation of its endothelium and connective tissue soon effects definite closure of the vessel, the effused blood is removed, the œdematous fluid is carried away by the lymphatics, and the vascular tissue recovers its tone. As for the coloring matter, the greater part of it remains in the connective-tissue spaces as a relic of the experiment, while eventually a part of it is carried by phagocytes to the nearest lymphatic gland.

Having thus proved that foreign masses floating in the blood could be so easily deposited in the tissues of the joint, it was but a step to the study of the fate of tubercle bacilli similarly introduced into the blood-current. Instead of injecting inert matter into the trachea, or directly into the vascular system, finely-divided tubercular matter was used. The same injuries as before were produced in the joints.

After varying periods the amimals either died of tuberculosis pulmonum or were killed. That the bacilli with which the blood was thus artificially infected were, like the granules of coloring matter, deposited in the injured joint-cavities at the site of hæmorrage was proved by the facts that tubercles sprang up only within the damaged joints and that blood-pigment was found within the tubercle cells.

Infection of a part having occurred at a point of minute hæmorrage, tubercles spring up in the immediate vicinity. In joints, the neighboring synovial membrane becomes vascularized, thickened, and red, and is eventually permeated by tubercular granulation tissue which has a cheesy, crumbly character.

Rarely does this process remain entirely local. For the infectious material is readily carried to remote parts of the joint cavity where a favorable point may be found for its development. Thus, several localities may be consecutively affected or the tuberculosis may become diffuse. But even in parts remote from terbercular foci, the synovial membrane exhibits a reddened vascular appearance, and without the presence of tubercles may eventually become covered with a layer of pale indolent granulations. Granulations of both kinds not infrequently fill the entire cavity of the joint and distend it till it gives to the touch a spongy resilient feeling that is useful as a diagnostic point.

It is these granulations which are so well-known to the clinical surgeon as the grayish gelatinous matter that represents the destroyed synovial membrane and causes the disease to be so frequently described as fungous, gelatinous, spongy, or hyperplastic granular arthritis.

Associated with such a synovitis it is not uncommon to notice the presence of morbid products within the joint. Clinically their appearance presents great variety. Their consideration is much facilitated by reference to their constituents, which may be divided into liquid and solid.

Of the liquid constituents the serous element is the predominant one, and when to this fibrin is added, we have a true sero-fibrinous effusion. Adding to the serum a quantity of solid tubercular matter in a state of coagulation necrosis, and an abundance of leucocytes which are usually, though not always, found in these exudations, we have a joint cavity filled with tubercular pus, an "*empyœma articuli.*"

Incidental hæmorrhages will be represented by the presence of red corpuscles or of blood pigment. The solid bodies observed in these joints are chiefly the result of the coagulation of fibrin into masses similar to rice bodies. They are usually deposited on the granulation layers, and become detached at a later period. They are especially observed in the form known as fibrous tuberculosis, in which *hydrops articuli* is very common.

While these active processes have been going on within the synovial cavity, the cartilage of the joint has played an entirely passive and subordinate part. Its avascular character forbids its becoming the primary seat of tubercular lesions, and it is only when infection has destroyed a part of the protecting synovialis that grave changes in it can occur. Thus, defects can be found in its substance, or only a local thinning. If the joint is filled with pus, a kind of maceration affects the surface of the exposed cartilage. As a result of chronic-tubercular inflammation, a metaplasia of the cartilage takes place, the cartilage superficially being transformed by vascularization into mucous membrane.

Consecutive to synovial tuberculosis bone affections must be regarded as either directly or indirectly the result of that process. In the former case the disease process acts immediately upon the bone tissue, producing eventually a resorption of the bone substance. But even where no infection has occurred a fatty atrophy of the bone takes place, due to the faulty nutrition of the parts, and therefore indirectly to the joint disease.

The periarticular tissues, in the more advanced form of the disease, are involved in

œdematous infiltration. The connective tissue takes on a coarse, fibrous, bacon-like character, while the skin acquires the well-known, smooth, shining, and pale appearance of *tumor albus*.

Tubercular Abscesses.—When, however, the para-articular tissue is reached by bacilli from the diseased joint, a condition is presented that is of great interest clinically. Since the synovial membrane may be regarded as a lymph sac, it is not unreasonable to suppose that bacilli might be carried by lymphatic vessels to the peri-articular tissues, just as we know that they are often carried to distant lymph glands. But the usually recognized method of infection is by direct outward extension of a tuberculosis of the articulating bones, or of the synovial membrane. When a synovitis tuberculosa occurs, in which there exists the tendency as above discussed to production of so-called pus, the synovial membrane is soon distended to its utmost capacity. Pressure atrophy occurs at that part of the capsule which is most imperfectly supported, and which is most weakened by morbid processes within. Rupture of the capsule relieves the pressure by permitting the escape of the fluid into the extra-articular tissue, and in many cases, collapse having taken place, the rent in the capsule is repaired by cicatrization. In this way cold abscesses often arise without the pathologist being able to find the exact point of origin. Hence it was formerly contended by many that they were often due to simple irritation. A space being formed by such a forcible means, it will naturally occur in that tissue whose mechanical resistance is least, namely, the loose connective tissue between muscles, membranes, viscera, and other structures. The newly invaded connective tissue is first covered by a layer of fibrin which is formed from the sero-fibrinous fluid. This forms a matrix for the development of tubercles. The original connective tissue below now rapidly proliferates, and speedily becomes thickened, and in this way a so-called pyogenic membrane is formed. Such an abscess may be distended till its own wall in turn ruptures, and discharges the tubercular débris, either into hitherto undisturbed connective tissue, into the outside air, or into some cavity or viscus of the body. The direction in which such abscesses spread is determined, therefore, by their point of origin, and by the relative resistance of the tissues in their vicinity.

The rupture of such an abscess into some of the important organs and cavities of the body may be followed by shock, embolism, general tubercular infection, or other dire consequence. Rupture into the external air is almost invariably followed by mixed infection of the abscess cavity, and by the ultimate formation of fistulæ leading into the joint.

Bone Tuberculosis.—In reviewing the subject of primary synovitis tuberculosa omission has been made of that form of synovitis observed in the joints of those who have died of general miliary tuberculosis. Miliary tuberculosis of bone, occurring under the same circumstances, is of equal interest to the student of pathology and equally unimportant to the clinical surgeon. In each case the constitutional nature of the disease and its speedy termination in death precludes local interference.

There are three forms of bone tuberculosis, however, which, on account of their frequency and intrinsic importance, as well as on account of the grave complications which they may superinduce, demand the earnest study of the clinical surgeon. The three forms to which I refer are tubercular osteomyelitis, and local intra-osseous tuberculosis, in the one case associated with the "wedge-formed" sequestrum, and in the other case occurring as a tubercular granulation mass.

The peculiar appearance of the wedge-formed sequestra led to suspicions long ago that their origin must be sought in embolism. It was in 1884 that Koenig added the weight of his opinion to previous suggestions, and in 1886, W. Müller published the result of a series of experiments undertaken to demonstrate the origin of bone tuberculosis. His most important experiments consisted in injecting into the nutrient arteries of rabbits' and goats' tibiæ a variable quantity of tubercular matter suspended in a very weak salt solution. The result was chiefly dependent upon three circumstances:

1. The relative quantity of tubercular matter and of salt solution.
2. The size of the masses suspended.
3. The proportionate number of bacilli in the material injected.

For if a relatively large mass of bacteria-bearing matter was lodged somewhere within a nutrient artery, it would naturally tend to infect a large mass of tissue corresponding to the area supplied by the vessel affected, and produce ultimate necrosis of a wedge-shaped mass of bone. But when the relatively large nutrient artery of such a bone as a phalanx or a carpal or tarsal bone was plugged near the entrance of the vessel into the bone, obviously an osteomyelitis tuberculosa would occur—that is, the whole of the bone would be infected. Supposing, on the other hand, that the masses used were minute enough to reach some of the smallest branches of the nutrient artery, it is shown that a mass of tubercular granulations is produced that involves directly in necrosis only very small masses of bone. Proceeding still further, a capillary lodgment of bacilli results in a miliary tuberculosis, and a part of the injection may even pass through the capillaries into the veins and thence infect the system at large.

The chief interest, therefore, lies in the study of the "wedge" and "granulation-mass" forms. Although the "wedge" form is likened to the hæmorrhagic infarct, its nature is essentially different. For here the assumption of the existence of terminal arteries, as averred by Cohnheim, is entirely unnecessary. When a tubercular mass has lodged in an artery supplying a considerable area of bone, the nutrition of that area is of course much impaired. The plug of infected material immediately sets up an endarteritis tuberculosa which ultimately results in the destruction of the arterial wall and infection of the para-vascular tissue. Simultaneously tubercular granulations spring up at the border line between the healthy tissue and that supplied by the affected artery. In this way sequestration is early effected. The new-formed connective tissue shows a tendency to contract and thus to limit the spread of the disease throughout the neighboring bone tissue, which usually beeomes somewhat porotic. The limiting layer of granulations has, however, an osteoclastic action upon the bone within it, which, however, even in the most favorable cases, is probably not completely resorbed (Koenig), thus accounting for the occurrence of relapses after recovery seemed perfect. At the same time this connective-tissue layer firmly binds the sequestrum to the healthy bone tissue.

In children the cartilage of the joint, in case the artery involved lies near one, may be part of the sequestrum; but its involvment is usually secondary. For infection by continuity of tissue is easily effected in this direction along the Haversian canals. The cartilage succumbs to the action of the disease, and the capsule of the joint is exposed. The sequestrum may form beneath the periosteum, and in that case the exit of the tubercular matter takes place through that member into the soft tissues without.

Embolism of a smaller artery than that mentioned does not result in the formation of large sequestra, but in the formation of a granulation mass, in which, however, small particles of bone may be found. An endarteritis is the beginning of the local disease process, after embolism has occurred. The bacilli having gained access to the surrounding tissues, tubercular granulations spring up and form a focus within the bone. The tendency may be toward rapid softening and pus formation, or toward peripheral cicatrization. This form also usually occurs in the spongy bone near the periphery, and may consequently infect either the periosteum or a joint cavity.

Synovitis tuberculosa, produced either by infection from a sequestrum focus or from a granulation-mass, partakes of the morbid character and tendency of the original focus. The pathological anatomy of the synovitis, moreover, varies in but slight particulars from that which is observed in synovitis arising from direct hæmorrhagic infection.

The energy of the tissues is manifested under some circumstances not only by the limitation of the progress of the disease, but also by the formation of osteophytes and by the thickening and hardening of pre-existent bone.

884 West Madison Street.

